# Mass spectrometric analysis of prefrontal cortex proteins in schizophrenia and bipolar disorder

**DOI:** 10.1186/2193-1801-1-3

**Published:** 2012-04-11

**Authors:** Shaheen E Lakhan

**Affiliations:** 1 Global Neuroscience Initiative Foundation, Los Angeles, CA, USA

## Abstract

**Background:**

Schizophrenia and bipolar disorder are the two most serious and debilitating neuropsychiatric disorders that share many characteristics, both symptomatic and epidemiological. There has yet to be a single diagnostic biomarker discovered for schizophrenia and bipolar disorder. Proteomics holds promise in elucidating the pathophysiology of these neuropsychiatric disorders from each other and healthy individuals.

**Findings:**

Postmortem prefrontal cortex tissue from schizophrenia, bipolar disorder, and psychiatric-free controls (n = 35 in each group) were subject to SELDI-TOF-MS protein profiling. There were 13 protein peaks distinguishing schizophrenia versus control and 15 in bipolar versus control. Using a predictor set of 10 peaks for each comparison, 73% prediction accuracy (*p* = 2.3×10^−4^) was achieved. Three peaks were in common between schizophrenia and bipolar disorder.

**Conclusions:**

This pilot study found protein profiles that distinguished schizophrenia and bipolar patients from controls and notably from each other. Identifying and characterizing the proteins in this study may elucidate neuropsychiatric phenotypes and uncover therapeutic targets. Further, applying class prediction bioinformatics may allow the clinician to differentiate the two phenotypes by profiling CSF or even serum.

## Findings

### Background

Schizophrenia and bipolar disorder are the two most serious and debilitating neuropsychiatric disorders that share many characteristics, both symptomatic and epidemiological. Each disorder affects roughly 1% of the population, has equal risk across gender, persists through a patient’s lifespan, and often affects patients after puberty and before 25 years of age. In addition, the course of illness is episodic in both disorders and places the sufferer at an increased risk of suicide. Genetic studies have mapped many susceptibility loci common to both diseases as well as large chromosomal aberrations (Gogos and Gerber [2006]; Pearlson and Folley [2008]). The prefrontal cortex (PFC) has been identified as a prominent site of dysfunction based on substantial neuroimaging, clinical, and postmortem studies, and microarray studies (Liemburg et al. [2012]; Teffer and Semendeferi [2012]; Volpe et al. [2012]). However, gene expression data do not consistently correlate with protein expressions and cannot identify post-transcriptional and post-translational modifications, major modulators of protein function (Ideker et al. [2001]; Lakhan and Vieira [2009]; Gygi et al. [1999]). Proteomic approaches are able to characterize post-translational modifications, a method by which the cell dynamically and quickly modifies protein function and regulates both creation and degradation in response to cellular perturbations (e.g. disease provocation) (see (Lakhan [2006]) for review).

There has yet to be a single diagnostic biomarker discovered for schizophrenia and bipolar disorder. While clinical biomarkers have tremendous diagnostic benefits, analyzing proteins from brain tissues of disease phenotypes is a superior initial approach to reveal differentially expressed proteins that may elucidate schizophrenia and bipolar disorder etiology and contribute to the understanding of neuropathogenesis and molecular psychiatry.

This pilot study demonstrates the possibility of accurately and sensitively distinguishing schizophrenia from bipolar disorder based on proteomic level data. Rather than using CSF or serum where potential pathogenic-revealing biomarkers are masked by common plasma proteins (e.g. platelet factors), human PFC tissues was subject to proteomic profiling to yield protein biomarkers.

## Methods

Postmortem prefrontal cortex (BA10) were dissected from patients with schizophrenia, bipolar disorder, and non-psychiatric control subjects. Each group consisted of 35 subjects. Diagnosis was made according to the Diagnostic and Statistical Manual of Mental Disorders, 4th Edition (DSM-IV).

Brain tissues samples were cut into 50 mg pieces. The cut samples were incubated with lysis buffer (urea, CHAPS, and DTT). The samples underwent tissue homogenization via mechanical disruption and centrifugation. The supernatant was extracted containing the tissue lysate protein content. All samples are stored at -80˚C until mass spectrometry application.

Tissue lysates were normalized to total protein concentration using lysis buffer. Tissue lysates were unfractionated. All samples were automatically and simultaneously processed in duplicate on Ciphergen ProteinChip arrays with the special chromatographic surfaces immobilized metal affinity capture (IMAC30). Briefly, the arrays were generally activated with proper solution, treated with washing/binding buffer, co-incubated with sample, mixed for several cycles to allow for protein binding to surface. Subsequently, unbound proteins were washed away with wash/binding buffer. Then, the arrays were concurrently treated with a saturated sinapinic acid solution (the energy absorbent molecule) and allowed to dry.

Arrays were analyzed with the Ciphergen ProteinChip Reader for surface enhanced laser desorption/ionization time-of-flight mass spectrometry (SELDI-TOF-MS) protein profiling. Spectra were normalized to the total ion current and the baseline was subtracted. Peak labeling and clustering were performed using the Ciphergen Biomarker Wizard, exported into a worksheet, and intensity values for each peak averaged for duplicate samples.

The algorithm structural pattern was employed using a localization analysis by sequential histogram (SPLASH) (Califano [2000]), a supervised method designed to discover patterns of multivariate associations in gene and protein expression data (Lepre et al. [2004]). Class prediction was done using the weighted voting algorithms where the informative peaks in the training set are used to perform leave-one-out cross validation (Golub et al. [1999]). The process started with two groups (e.g. schizophrenia and control) and a set of features (i.e. informative protein peaks). A sample was left out and a predictor set of peaks that differentiate between the two groups was built. The sample that was left out was then classified as one of the two groups using the predictive peaks. This was cycled through all samples individually. The accuracy of the predictor was assessed by the total number of correct predictions. The p-value for the predictor accuracy is calculated using Fisher’s test.

This biostatistical approach was carried out on two comparisons: 1) schizophrenia vs. control and 2) bipolar vs. control. The identified independent patterns were provided by 60–70% of the samples in a given phenotype.

## Results

**Figure 1:**
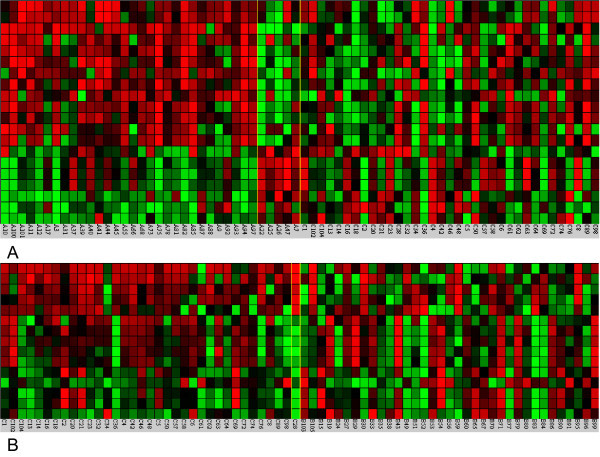
**Heatmap of proteomic patterns a) schizophrenia vs. control, and b) bipolar disorder vs. control.** The samples are broken in three groups, separated by vertical yellow lines that are shown from left to right. These three groups of samples are: samples in the phenotype where the pattern appears, samples in the phenotype where the pattern does not appear, and samples used as the control. Green: underexpressed proteins. Red: overexpressed proteins.

There were 13 protein peaks representing consistent patterns in schizophrenia vs. control and 15 in bipolar vs. control (see Figure 1). The discovered patterns were then used to perform leave-one-out cross validation in the relevant phenotypes. Namely, out of the 13 peaks that differentiate schizophrenia from control, a predictor set of 10 peaks was used and obtained 73% accuracy in the prediction. The results for bipolar vs. control were the same with 73% prediction accuracy using 10 peaks selected out of 15 informative peaks. In both cases, the significance of the prediction accuracy assessed by Fisher’s test was at a p-value of 2.3 × 10^−4^.

**Figure 2:**
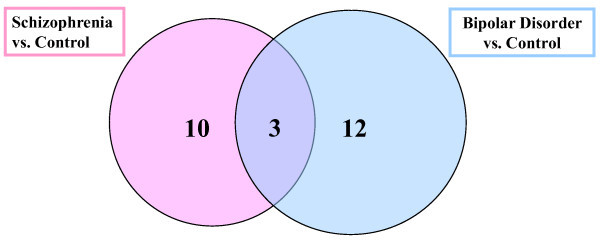
**Venn diagram of differentially expressed proteins (biomarkers).** Three proteins are shared biomarkers of the 13 and 15 total biomarkers discovered by Schizophrenia vs. Control and Bipolar Disorder vs. Control analyses, respectively.

The overlap between the 13 and 15 informative protein peaks that differentiate schizophrenia and bipolar disorder, respectively, from controls were also investigated. The results are shown in Figure 2, where the overlap between these peaks is 3.

## Discussion

The experimental design presented in this study demonstrates preliminary neuropathological investigation and revelation of protein profiles in schizophrenia and bipolar disorder, both shared and exclusive to each diagnosis. Three protein peaks were found to be differentially expressed in both schizophrenia and bipolar disorder from controls and, notably, 10 and 12 protein biomarker peaks were exclusive. While it is essential to identify similarities in schizophrenia and bipolar disorder, it is equally or more so important to classify the changes specific to a given neuropsychiatric condition. Where altered expression overlaps between disorders suggest a common pathology and perhaps attributed to the similar symptomatic profile, the exclusive changes may elucidate and distinguish the phenotype. Diagnostically, applying class prediction bioinformatics may allow the clinician to differentiate the two phenotypes by profiling serum or CSF. In addition, where drug discovery is dynamically evolving, direct proteomic data of identified and characterized biomarkers may be viable drug target proteins. Further investigations to sequence and further characterize the biomarker proteins are planned.

The vast majority of pharmaceutical agents target proteins. Inherently, direct study of diseased proteomes is the essential utility for drug discovery and clinical proteomics (Mikami et al. [2011]; Trist [2011]). In fact, it is believed that proteomics-based tests are likely to be to a large extent more predicative than genetic tests, which tend to be more non-specific (Wilson [2004]). The robust and high-throughput nature of mass spectrometry applications allows streamlined identification of new disease specific targets. In addition, schizophrenia and bipolar specific protein targets may customize pharmacotherapy and thereby augment the efficacy and decrease the toxicity potential of drugs.

Proteomics in the post-genomic era has the capability of characterizing macromolecules and their interactions, complexes, and networks. Ultimately, biomarker discovery, class prediction, and elucidation of schizophrenia and bipolar disorder neuropathogenesis are worthy goals with a large potential benefit and minimized risks.

## Abbreviations

BA10: Brodmann’s area 10; DSM IV: Diagnostic and Statistical Manual of Mental Disorders, 4th Edition; PFC: Prefrontal cortex; SELDI-TOF-MS: Surface enhanced laser desorption/ionization time-of-flight mass spectrometry; SPLASH: Structural pattern localization analysis by sequential histogram.

## Competing interests

The author declares that he has no competing interests.
